# HBsAg level defines different clinical phenotypes of HBeAg(−) chronic HBV infection related to HBV polymerase-specific CD8^+^ cell response quality

**DOI:** 10.3389/fimmu.2024.1352929

**Published:** 2024-03-13

**Authors:** Julia Peña-Asensio, Henar Calvo-Sánchez, Joaquín Miquel-Plaza, Eduardo Sanz-de-Villalobos, Alejandro González-Praetorius, Alberto Delgado-Fernandez, Miguel Torralba, Juan-Ramón Larrubia

**Affiliations:** ^1^ Department of Biology of Systems, University of Alcalá, Alcalá de Henares, Spain; ^2^ Translational Research Group in Cellular Immunology, Instituto de Investigación Sanitaria de Castilla La-Mancha (IDISCAM), Toledo, Spain; ^3^ Section of Gastroenterology, Guadalajara University Hospital, Guadalajara, Spain; ^4^ Department of Medicine and Medical Specialties, University of Alcalá, Alcalá de Henares, Spain; ^5^ Section of Microbiology, Guadalajara University Hospital, Guadalajara, Spain; ^6^ Service of Internal Medicine, Guadalajara University Hospital, Guadalajara, Spain

**Keywords:** HBV inactive carrier, functional HBV-specific CD8 + T-cell response, HBV immune control, liver fibrosis progression, HBsAg, HBV eAg(-) infection gray-zone

## Abstract

**Background:**

HBe-antigen(Ag)-negative chronic hepatitis B virus (HBV) infection is characterized by little liver fibrosis progression and vigorous HBV-multispecific CD8^+^ T-cell response.

**Aims:**

To assess whether HBsAg level could discriminate different HBeAg-negative chronic HBV infection subtypes with dissimilar quality of HBV-specific CD8^+^ T-cell response.

**Methods:**

We recruited 63 HBeAg-negative chronic HBV infection patients in which indirect markers of liver inflammation/fibrosis, portal pressure, viral load (VL), and HBV-specific CD8^+^ cell effector function were correlated with HBsAg level.

**Results:**

A positive linear trend between HBsAg level and APRI, liver stiffness (LS), liver transaminases, and HBV VL, and a negative correlation with platelet count were observed. Frequency of cases with HBV-specific CD8^+^ T-cell proliferation against at least two HBV epitopes was higher in HBsAg < 1,000 IU/ml group. CD8^+^ T-cell expansion after HBVpolymerase_456-63_-specific stimulation was impaired in HBsAg > 1,000 IU/ml group, while the response against HBVcore_18-27_ was preserved and response against envelope_183-91_ was nearly abolished, regardless of HBsAg level. Cases with preserved HBVpolymerase_456-63_ CD8^+^ cell response had lower LS/duration of infection and APRI/duration of infection rates. HBV-polymerase_456-63_-specific CD8^+^ T-cell proliferation intensity was negatively correlated with LS/years of infection ratio.

**Conclusion:**

HBsAg > 1,000 IU/ml HBeAg-negative chronic HBV infection group shows indirect data of higher degree of inflammation, liver stiffness, and fibrosis progression speed, which are related to an impaired HBV-polymerase-specific CD8^+^ T-cell response.

## 1 Introduction

Persistent hepatitis B virus (HBV) infection has different well-established phases depending on the relationship between the immune response and viral replication (1). Phase 3, classically termed “inactive carrier” and currently hepatitis B e-antigen (Ag) negative [HBeAg(−)] chronic HBV infection (2), is characterized by low HBV replication and an HBV-multispecific CD8^+^ T-cell response, linked to low liver inflammation and low fibrosis progression (3). The diagnosis of HBeAg-negative chronic HBV infection is based on long-term follow-up to assess the maintenance of low HBV replication and persistently normal alanine aminotransferase (ALT) level (2). However, the addition of hepatitis B surface (HBs) Ag in patient evaluation increases diagnostic accuracy (4). The subset with HBsAg > 1,000 IU/ml shows a higher percentage of cases with worse disease progression during long-term follow-up (5).

The presence of a functional HBV-multispecific CD8^+^ T-cell response has been correlated with development of HBV control and functional cure after nucleoside analogue treatment discontinuation in HBeAg(−) chronic hepatitis B (6). In that study, low HBsAg level was one of the predictive factors linked to restoration of a functional HBV-specific cytotoxic T-cell (CTL) response. In the same line of thought, HBsAg level could also be involved in the quality of the adaptive immune response in HBeAg-negative chronic HBV infection. Moreover, in low replicative chronic HBV infection, HBVpolymerase(pol)-specific CD8^+^ T cells showed a reduced expansion capacity compared to HBVcore-specific CTLs that was related to an imbalanced TCF1/BCL2 expression (7). Therefore, it could be the case that some of the considered HBeAg-negative chronic HBV infection with an elevated HBsAg level presented an impaired HBV-specific CTL response, which could be associated with worse disease progression, especially in those patients categorized in the “gray zone” (8). To assess this issue, we carried out a cross-sectional study in which we evaluated indirect parameters of liver inflammation, liver fibrosis, HBV replication, and functionality of peripheral HBV-specific CD8^+^ T-cell response as function of HBsAg level in a Mediterranean cohort of inactive carriers.

## 2 Material and methods

### 2.1 Study design

We carried out a cross-sectional analytical study. A total of 63 patients with HBeAg-negative chronic HBV infection were recruited from the Translational Hepatology Unit HBV database of the Guadalajara University Hospital (Spain) (**Table 1**). All enlisted cases were Caucasians, born in Mediterranean countries. We excluded patients co-infected with hepatitis C, hepatitis D, and human immunodeficiency virus; with alcohol intake of more than 40 g/day; had cirrhosis; or had body mass index (BMI) > 30 kg/m^2^. Clinical diagnosis was performed according to the European Association for the Study of the Liver (EASL) (2). Specifically, patients diagnosed with HBeAg-negative chronic HBV infection had to have a minimum 1 year follow-up with at least three assessments in the first year of follow-up with ALT ≤ 50 IU/ml and HBV DNA ≤ 20,000 IU/ml and liver stiffness (LS) ≤ 7 kPa. They should also have maintained an ALT value below the upper limit of normal and HBV DNA ≤ 20,000 IU/ml during the subsequent annual follow-up after diagnosis. At recruitment, we recorded sex at birth, age, country of origin, estimated moment of HBV transmission, source of infection, BMI, duration of follow-up, ALT (IU/ml), aspartate aminotransferase (AST) (IU/ml), HBV DNA (IU/ml), platelet count (×10^3^/μl), LS by transient hepatic elastography (kPa), AST to platelet ratio index (APRI), and HBsAg (IU/ml) level. The rate of progression of liver fibrosis was estimated by APRI and by LS (kPa) to duration of infection ratio. The timepoint of infection was estimated by the time of exposure to an HBV transmission risk factor. In the absence of a clear risk factor, it was assumed that the infection was acquired by horizontal transmission during childhood by intramuscular injection and bruises playing with other HBV-positive children, as it was common in the Mediterranean basin (9). The number of HBV DNA test performed during follow-up and the number with a result above 2,000 IU/ml were retrospectively recorded. Heparinized blood and serum samples were drawn for immunological and virological testing. In human leukocyte antigen (HLA)-A2^+^ patients, HBV-specific CD8^+^ T-cell response against core, pol, and envelope (env) epitopes were assessed. Retrospectively, ALT (IU/ml), AST (IU/ml), APRI, HBV DNA (IU/ml), and platelet count (×10^3^/μl) at diagnosis were registered.

**Table 1:** Clinical features of HBeAg-negative chronic HBV infection patients enrolled in the study, according to HBsAg level.

	HBsAg level (IU/ml)			p-value
	< 300 (n= 12)	300–1,000 (n=13)	>1,000 (n=38)	
**Age (years)**	58 (17)	49 (23)	49 (16)	NS^†^
**Sex (male %)**	42	23	47	NS^‡^
**Duration of infection (years)**	49 (19)	40 (23)	44 (12)	NS^†^
**Follow-up (years)**	7.5 (7)	5 (8)	4.5 (8)	NS^†^
**HLA-A2 (%)**	83	39	44	**0.040^‡^**
**Origin (%)**				NS^‡^
**• Spanish** **• Romanian** **• Other**	67 17 16	40 50 10	61 25 11	
**Source of infection (%)**				NS^‡^
**• Horizontal** **• Parenteral** **• Vertical** **• Other**	58 33 0 8	61 23 7 7	79 18 3 0	
**Alcohol intake < 40 g/day (%)**	100	100	100	NS^‡^
**HBsAg (Log IU/ml)**	0.9 (3)	2.7 (0.2)	3.6 (0.8)	**<0.001^†^**
**HBV genotype (%)**				NS^‡^
**• A** **• D**	0 100	15 85	35 65	
**Body mass index (kg/m^2^)**	25.4 (4.1)	23.1 (5.4)	25.4 (4.5)	NS^†^
Analytical data at diagnosis				
**• ALT (IU/ml)**	21 (10)	18 (15)	23 (14)	NS^†^
**• AST (IU/ml)**	19 (15)	19 (10)	22 (10)	NS^†^
**• APRI**	0.22 (0.14)	0.22 (0.23)	0.25 (0.17)	NS^†^
**• HBV DNA (Log IU/ml)**	2.1 (1.5)	2.9 (1.4)	2.7 (1)	NS^†^
**• Platelet count (x10^3^/ml)**	224 (62)	209 (81)	211 (65)	NS^†^

Data are expressed as frequency distribution for categorical variables and as median plus interquartile range for quantitative variables. ALT, alanine aminotransferase; APRI, AST to platelet ratio index; AST, aspartate aminotransferase; HBsAg, HBV surface antigen; HBV, hepatitis B virus; HLA, human leukocyte antigen; N.S., non-significant. ^†^Wilcoxon test. ^‡^Chi-square test. Bold values mean that the p value is significant.

The study protocol was approved by the Research Ethical Committee of the Guadalajara University Hospital (Spain). All the patients enrolled in the study gave written informed consent. The study protocol was in accordance with the ethical guidelines of the 1975 Declaration of Helsinki.

### 2.2 Human leukocyte antigen-A2

Screening for HLA-A2 haplotype was performed by staining with anti-HLA-A2 fluorescein 5-isothiocyanate monoclonal antibody (mAb), (Clone BB7.2) (Biolegend, San Diego, CA). We did not perform a four-digit HLA-A2 analysis, since most of the HLA-A2 Spanish patients are HLA*A2:01 (10) and therefore suitable for study with HLA-A2:01/peptide pentameric complexes (pentamers).

### 2.3 Synthetic peptides and pentamers

HLA-A2-restricted peptides corresponding to the HBV-env_183-91_, HBV-core_18-27_, and HBV-pol_455-63_ regions and phycoerythrin-(PE)-conjugated HLA-A2-pentamers loaded with the same HBV peptides were purchased from ProImmune Ltd. (Oxford, UK).

### 2.4 HBV-specific CD8^+^ T-cell detection

To quantify HBV-specific CD8^+^ T cells, 1×10^6^ peripheral blood mononuclear cells (PBMCs) were stained for 10 min at room temperature at saturating concentration of PE-labeled pentamers in Roswell Park Memorial Institute (RPMI) 1640 medium plus 10% heat-inactivated fetal bovine serum (HI-FBS). Cells were washed in phosphate-buffered saline and then incubated at 4°C for 20 min with saturating concentrations of directly conjugated anti-CD8-Peridinin-Chlorophyll-Protein (PerCP) mAb (Clone SK1) (Biolegend, San Diego, CA) and anti-CD3-Alexa Fluor 647 (UCHT1) (Biolegend, San Diego, CA). Subsequently after washing, stained cells were acquired on a Becton Dickinson FACS^®^ Calibur flow cytometer and analyzed on FlowJo™ software v10 (Becton Dickinson Bioscience, San Jose, CA). A positive detection of directly *ex vivo* HBV-specific CD8^+^ T cells was considered when a cluster of pentamer^+^/CD8^+^ cells higher than 0.02% out of the total CD8^+^ cells was detected, as previously described (3).

### 2.5 Ag-specific *in vitro* challenge of HBV-specific CD8^+^ T cells

PBMCs were resuspended at a concentration of 1×10^6^/ml in RPMI 1640 medium plus 10% HI-FBS. Cells were stimulated with 1 μM of one of the different HBV peptides, in a 96-well plate. Recombinant interleukin-2 (25 IU/ml) was added on day 2 of culture, and cells were analyzed 10 days after *in vitro* challenge on FlowJo™ software v10 (Becton Dickinson Bioscience, San Jose, CA) after acquisition on a Becton Dickinson FACS^®^ Calibur flow cytometer. A positive expansion was considered when pentamer^+^/CD8^+^ cells were found in a cluster shape, and the frequency of positive cells was at least 0.1% out of total CD8^+^ cells with a minimum of 50 detectable dots.

### 2.6 Interferon-γ and tumor necrosis factor-α immunoassay

For quantitative determination of interferon-(IFN)γ and tumor necrosis factor-(TNF)α concentrations in cell culture supernatants after restimulation for 4 h with the specific peptide, commercial enzyme-linked immunosorbent assay (ELISA) kits (Quantikine^®^ ELISA human IFNγ Immunoassay and Quantikine^®^ ELISA human TNFα Immunoassay; R&D Systems, Minneapolis, MN) were used, according to the manufacturer’s instructions. The lower level of detection was 0.149 ρg/ml for IFNγ and 2.09 ρg/ml for TNFα. Our group previously verified that this analytical strategy could detect cytokine secretion by HBV-specific CD8^+^ T cells after specific expansion, by demonstrating a significant positive correlation between the type-I cytokine level on culture supernatant tested by ELISA and the fluorescence intensity after intracellular cytokine staining assessed by flow cytometry on HBV-specific CD8^+^ T cells after 10 days Ag-specific *in vitro* challenge (6).

### 2.7 Viral serum parameters, aminotransferase level and platelet count

ALT, AST, HBeAg, and anti-HBe were performed by standard immunoassay. Serum HBV DNA was measured by real-time PCR using the Cobas^®^ 6800 system (Roche Diagnostics, Manheim, Germany; LLQ <10 IU/ml). HBsAg level was determined by ARCHITECT^®^ HBsAg assay (Abbott Laboratories, Chicago, IL, USA) with a lower limit of detection of <0.13 IU/ml. HBV genotype was determined by direct sequencing and phylogenetic analysis of a fragment of 1,137 bp of the viral polymerase (nucleotides 108–1244, GenBank accession number NC003977) as described by Tong et al. (11). Platelet level was quantified using an automatized hematology analyzer. The mean percentage change in HBsAg level per year of follow-up respect to baseline level was recorded.

### 2.8 Liver fibrosis assessment

APRI value was calculated as [(AST level/AST upper limit of normal)/platelet count (×10^3^/μL)] × 100. Liver fibrosis was also estimated by transient hepatic elastography with FibroScan-402^®^ device (Echosens, France).

### 2.9 Statistical analysis

Quantitative and categorical variables are summarized as median plus interquartile range (IQR) and as frequency distribution, respectively. Chi-square, Mann–Whitney U, Wilconxon, and Spearman’s correlation tests were employed when appropriate. All tests were two-tailed and with a significance level p<0.05. Statistical analyses were performed with SPSS version 25.0 software (IBM, Chicago, IL).

## 3 Results

### 3.1 Different clinical phenotypes in HBeAg(−) chronic HBV infection according to HBsAg level

To assess whether HBsAg level correlated with different degrees of liver inflammation and fibrosis in HBeAg-negative chronic HBV infection, we performed a cross-sectional study recruiting a Mediterranean cohort of 63 HBV-inactive carriers. This cohort had persistently had HBV DNA level ≤ 20,000 IU/ml and ALT ≤ 50 IU/ml during the first-year follow-up and were followed up for a median time of 5 years (IQR 7) before recruitment to the study. The level of liver fibrosis, inflammation, and portal pressure were estimated indirectly at the end of follow-up. This cohort was split into three groups according to the HBsAg level at the time of recruitment for this study. The demographic, virological, and clinical features of the groups are shown in **Table 1**. HBV DNA level allowed genotyping in one-third of patients, being in all tested cases genotype D or A.

**Figure 1:**
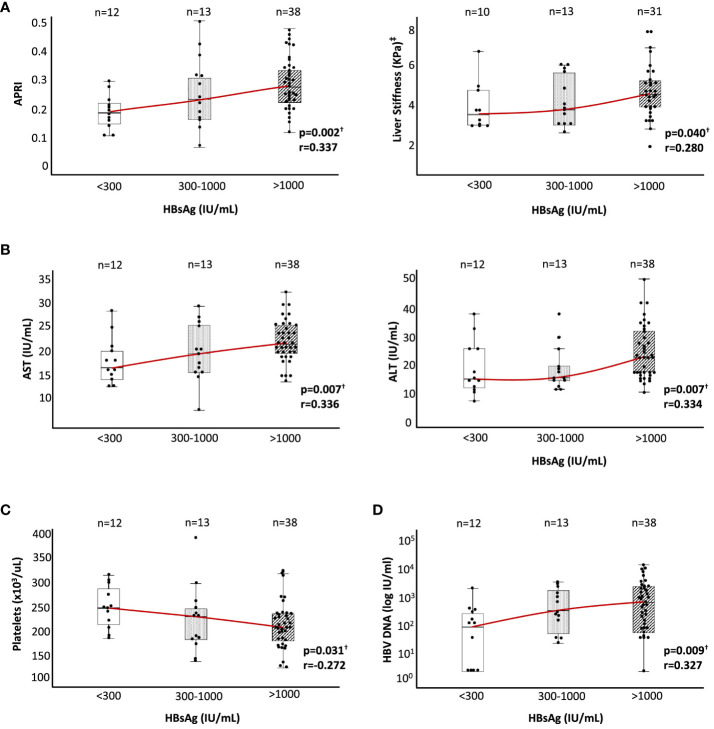
Liver inflammation, liver stiffness, platelet count, and HBV DNA level according to HBsAg level in HBeAg(−) chronic HBV infection. According to HBsAg level at recruitment, box plot graphs describing the distributions of **(A)** APRI and liver stiffness by hepatic transient elastography (kPa), **(B)** ALT and AST, **(C)** platelet count, and **(D)** HBV DNA level. ALT, alanine aminotransferase; APRI, AST to platelet ratio index; AST, aspartate aminotransferase; HBsAg, hepatitis B surface antigen; HBV, hepatitis B virus. ^†^Spearman correlation test. ^‡^In nine cases, liver stiffness data were not available at the time of recruitment.

We observed a significant positive correlation between HBsAg level, and the degree of liver fibrosis at the end of follow-up, estimated by APRI score and LS by transient liver elastography, although all groups displayed values within the normal range. The median APRI score in the HBsAg > 1,000 IU/ml group was 50% and 22% higher than in the HBsAg < 300 IU/ml and HBsAg 300–1,000 IU/ml cohorts, respectively. Similarly, the median kPa value of LS was ≈20%–30% higher in the high HBsAg level group relative to the two lower HBsAg level groups (**Figure 1A**).

The degree of liver inflammation estimated by transaminase level also showed a positive correlation with the HBsAg level at the end of follow-up, although all the values were within the normal range as was the case with liver fibrosis. The median value of ALT and AST increased by ≈50% and ≈30% in the group with high HBsAg level regarding the two other groups with lower HBsAg level (**Figure 1B**). We also checked the platelet count as an indirect indicator of portal pressure level. In this case, we observed a significant negative correlation between HBsAg and platelet levels, although again the values obtained were within the normal range. Platelet count decreased by 20% in the high HBsAg level group with respect to the low HBsAg level group, with an intermediate value in the HBsAg level cohort of 300–1,000 IU/ml (**Figure 1C**). Finally, we also observed a significant positive correlation between HBsAg and HBV DNA levels, with a ≈3-fold higher median value in the intermediate HBsAg level group and ≈8-fold higher in the high HBsAg level group with respect to the low HBsAg level cohort (**Figure 1D**).

### 3.2 HBV-specific CD8^+^ T-cell response according to HBsAg level

**Figure 2:**
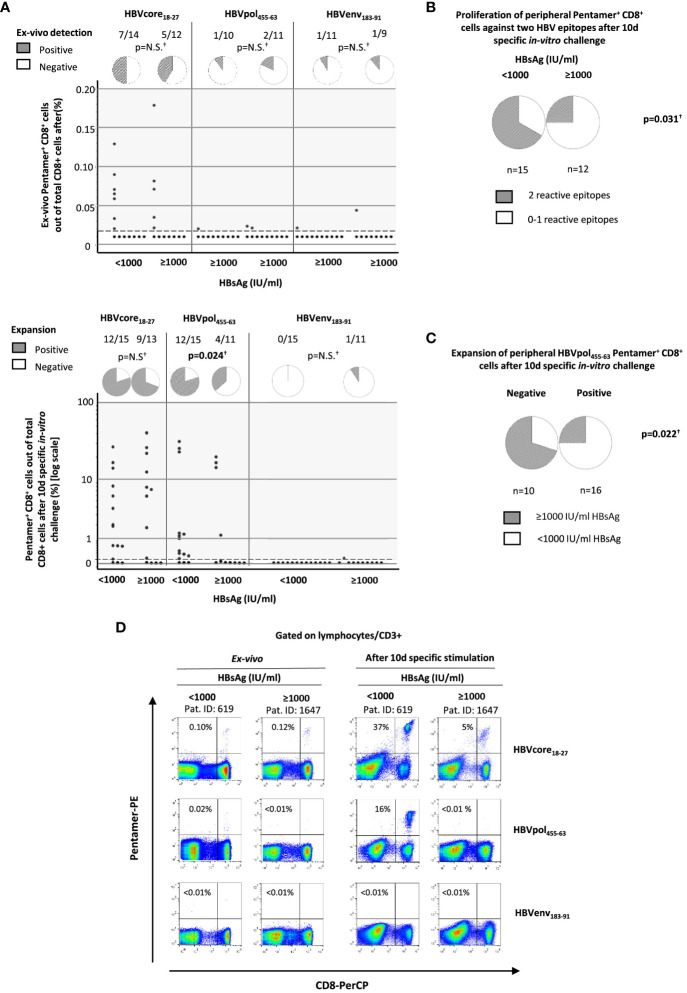
Quantification of peripheral HBV-specific CD8 T cells directly *ex vivo* and after 10-day Ag-specific *in vitro* challenge in HLA-A2^+^ subjects with HBeAg(−) chronic HBV infection according to HBsAg level. According to HBsAg level at recruitment, HBV-specific CD8^+^ T-cell response against three HLA-A2 restricted epitopes (core_18-27_, pol_465-63_, and env_183-91_) was analyzed by pentameric staining. **(A)** Dot plots showing the frequency of pentamer^+^ CD8^+^ T cells out of total CD8^+^ cells directly *ex vivo* and after Ag-specific stimulation and pie charts depicting the frequency of cases with directly *ex vivo* and after Ag-specific *in vitro* challenge detectable pentamer-binding CD8^+^ T cells. Each dot is one HBV-specific CD8+ T-cell population from one donor. **(B)** Frequency of cases with HBV-specific CD8^+^ T cells reactive against to at least two different HBV epitopes. **(C)** Percentage of cases with high and low HBsAg levels according to the presence or absence of reactive HBVpol_455-63_-specific CD8^+^ T cells. **(D)** Representative FACS Calibur BD™ dot plots showing the frequency of pentamer-binding CD8^+^ T cells directly *ex vivo* and after 10-day Ag-specific stimulation. The figure in each dot plot represents the frequency of pentamer-binding CD8^+^ T cells out of the total CD8^+^ T cells. Env, envelope; HBsAg, hepatitis B surface antigen; HBV, hepatitis B virus; N.S., non-significant; PE, phycoerythrin; Pent, pentamer; PerCP, Peridinin-Chlorophyll-Protein; pol, polymerase. ^†^Chi-square test.

To check whether the quality of immune response could be related to the different clinical phenotypes observed in HBeAg-negative chronic HBV infection, in the 28 HLA-A2^+^ patients of the study cohort, we quantified the *ex vivo* frequency and proliferation ability after Ag encounter of HBV-specific CD8^+^ T cells. Due to the sample size, we pooled the two lowest HBsAg level groups into the same group, thus comparing the HBV-specific CD8^+^ T-cell response between cases with HBsAg level below and above 1,000 IU/ml. Directly *ex vivo*, we observed in both groups a similar frequency of cases with detectable CD8^+^ T cells against HBVcore_18-27_ (50% vs. 42%), HBVpol_455-63_ (10% vs. 18%), and HBVenv_183-91_ (9% vs. 11%) epitopes (**Figures 2A, D** and **Table 2**). After 10-day Ag-specific *in vitro* challenge, the proliferation ability of HBVenv_183-91_-pentamer^+^ CD8 T cells was abrogated in most cases in both groups (0% vs. 9%). The expansion capacity of HBVcore_18-27_-pentamer^+^ CD8^+^ T cells was preserved in most cases, regardless of HBsAg level (80% vs. 69%), whereas the proliferation ability of HBVpol_455-63_-penatmer-biding CD8^+^ T cells was significantly more frequent in the group with HBsAg level <1,000 IU/ml (80%) compared to the cohort with HBsAg > 1,000 IU/ml (36%) (**Figures 2A, D** and **Table 2**). The frequency of cases with reactivity against at least two epitopes was higher in the low HBsAg level group (67% vs. 25%) (**Figure 2B** and **Table 2**), and this fact was explained by the difference in the positive expansion of HBVpol_455-63_-binding CD8^+^ T cells between the two HBsAg level groups (**Figure 2C** and **Table 2**).

**Table 2:** Frequency of HBV-specific CD8^+^ T cells directly *ex vivo* and after 10-day Ag-specific *in vitro* challenge in the cohort of HLA-A2^+^ HBeAg-negative chronic HBV infection patients according to HBsAg level.

		% HBV-specific CD8^+^ cells out of total CD8^+^ cells directly *ex vivo*			% HBV-specific CD8^+^ cells out of total CD8^+^ cells after 10-day Ag-specific *in vitro* challenge		
HBsAg>1,000 IU/ml							
Patient ID	logHBsAg (IU/ml)	Core_18-27_	Pol_455-63_	Env_183-91_	Core_18-27_	Pol_455-63_	Env_183-91_
329	4.42	**0.08**	0.00	0.00	**26.50**	0.00	0.03
1384	4.16	**0.18**	0.01	N.D	**40.80**	**14.50**	0.00
1558	4.12	**0.07**	**0.02**	N.D	**1.76**	**16.80**	N.D
929	4.04	**0.03**	0.00	0.00	**22.40**	**20.10**	0.00
1299	3.99	0.00	0.00	0.00	**0.13**	0.00	0.00
1608	3.86	N.D	N.D	N.D	0.01	N.D	N.D
927	3.62	0.00	N.D	0.01	0.00	N.D	0.00
934	3.57	0.01	**0.02**	0.00	0.00	**1.20**	0.03
1647	3.49	**0.12**	0.01	0.00	**5.03**	0.00	0.00
307	3.43	0.00	0.00	0.00	0.00	0.01	**0.16**
1574	3.36	0.01	0.00	N.D	**12.60**	0.01	0.08
594	3.31	0.00	0.00	0.00	**7.28**	0.00	0.00
1536	3.19	0.01	0.00	**0.04**	**7.79**	0.00	0.09
**% of Positive cases**		5/12 (**42%**)	2/11 (**18%**)	1/9 (**11%**)	9/13 (**69%**)	4/11 (**36%)**	1/11 (9**%**)
HBsAg<1,000 IU/ml							
Patient ID	logHBsAg (IU/ml)	Core_18-27_	Pol_455-63_	Env_183-91_	Core_18-27_	Pol_455-63_	Env_183-91_
1534	2.93	0.00	N.D	0.01	.00	**1.18**	0.00
1025	2.87	0.01	0.01	0.00	**2.01**	**1.36**	0.00
227	2.76	0.01	N.D	0.00	**0.62**	**.42**	0.00
1612	2.65	0.01	0.00	0.00	**3.80**	**0.33**	0.05
1494	2.63	**0.13**	0.01	0.00	**14.28**	**.23**	0.00
670	2.40	N.D	N.D	N.D	**0.62**	0.02	0.00
1562	2.38	**0.07**	0.00	N.D	**0.12**	**25.90**	0.05
626	2.37	**0.06**	0.00	**0.02**	**8.11**	**0.14**	0.00
619	2.35	**0.10**	**0.02**	0.00	**37.10**	**16.33**	0.00
545	1.20	**0.07**	N.D	0.00	**16.90**	**1.03**	0.03
982	.09	0.00	0.00	0.00	0.06	**1.31**	0.00
677	-.28	0.00	0.01	0.00	**1.78**	0.00	0.02
1318	-.74	**0.03**	N.D	0.00	**0.65**	**0.29**	0.00
687	-1.40	**0.02**	0.00	N.D	**5.86**	**31.70**	0.04
367	-2.00	0.01	0.01	N.D	0.05	0.03	0.00
**% of Positive cases**		7/14 (**50%**)	1/10 (**10%**)	1/11 (**9%**)	12/15 (**80%**)	12/15 (**80%**)	0/15 (**0%**)

The figures in bold show the cases with directly ex vivo positive detection or positive expansion after 10-day Ag-specific stimulation of CD8^+^/Pentamer^+^ cells. Directly ex vivo positive detection was considered when a cluster of CD8^+^/Pentamer^+^ cells higher than 0.02% of total CD8^+^ T cells was observed. After 10-day Ag-specific in vitro challenge, a positive expansion was considered when a cluster of CD8^+^/Pentamer^+^ cells higher than 0.1% out of total CD8+ cells was detected. Env, envelope; ID, identification; N.D., not done; Pol, polymerase.

**Figure 3:**
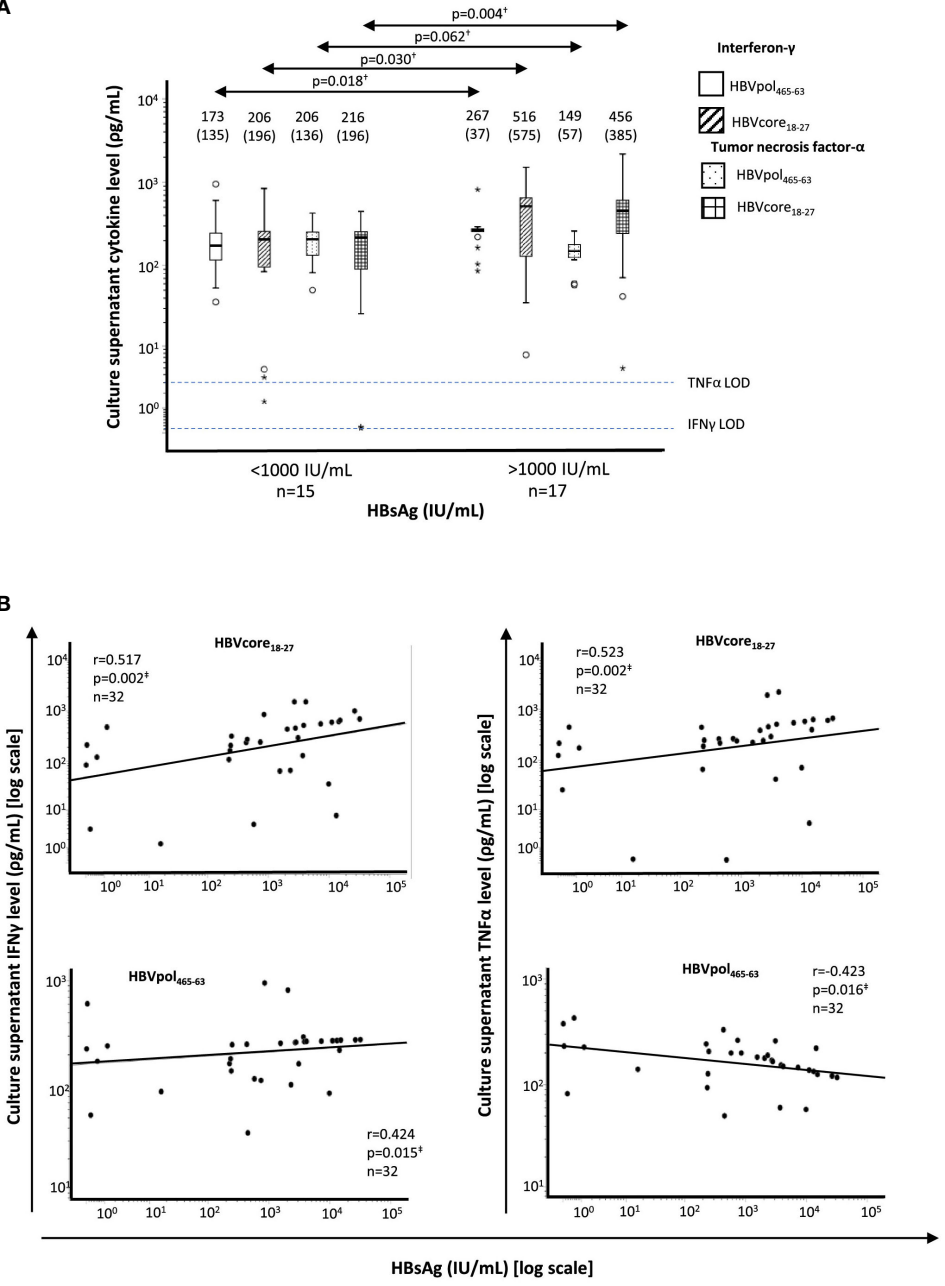
Correlation between HBsAg level and supernatant cytokine level after 10-day Ag-specific *in vitro* challenge of HBV-specific CD8^+^ T cells from HLA-A2^+^ HBeAg(−) chronic HBV infection patients. **(A)** Boxplots showing the interferon-γ and tumor necrosis factor-α concentration in T-cell culture supernatant after 10-day Ag-specific *in vitro* challenge, tested by commercial enzyme-linked immunosorbent assay. The figures on top of the boxplots represent the median cytokine value and the interquartile range between brackets. The horizontal dashed lines represent the cytokine limit of detection. **(B)** Scatterplot depicting the correlation between HBsAg level and the type-I cytokine level on culture supernatants of T-cell cultures after 10-day Ag-specific *in vitro* challenge. HBsAg, hepatitis B surface antigen; IFN, interferon; LOD, limit of detection; pol, polymerase; TNF, tumor necrosis factor. ^†^Mann–Whitney U test. ^‡^Spearman correlation test. The symbol "●" stands for outlier and the symbol "*" stands for extreme value.

In all HLA-A2^+^ patients, after a short-term Ag-specific restimulation on day 10 of culture, we collected culture supernatants to check the levels of IFNγ and TNFα in the two study groups. We observed a higher IFNγ and TNFα levels in HBVcore_18-27_ cultures from patients with HBsAg > 1,000 IU/ml. Nevertheless, although IFNγ did increase, TNFα level decreased in HBVpol_465-63_ cultures from HBsAg > 1,000 IU/ml group (**Figure 3A**). Moreover, we observed a positive correlation between HBsAg level and IFNγ concentration in both HBVcore_18-27_ and HBVpol_455-63_ cultures and a positive correlation with TNFα concentration in HBVcore_18-27_ cultures but negative correlation in HBVpol_455-63_ cultures (**Figure 3B**). These data should be taken cautiously, since they were obtained after a 10-day peptide-pulsed culture, and therefore, we cannot assure they come from precursor Ag-specific CD8^+^ T cells or they come from other sources such as reprogrammed cells or from bystander activation.

### 3.3 HBVpol_455-63_-specific CD8 T-cell response and liver fibrosis progression

**Figure 4:**
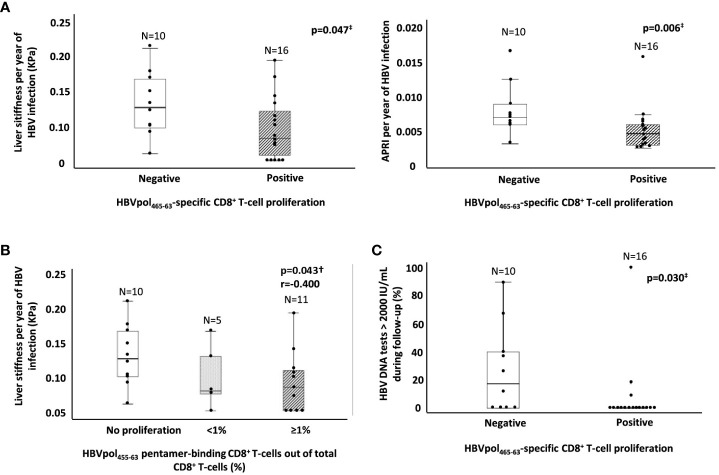
Ratio of estimated fibrosis progression and HBV DNA replication peaks during follow-up as a function of the presence of a reactive HBVpol-specific CD8^+^ T cells. **(A)** Boxplot graphs showing the distribution of both APRI score and liver stiffness per year of infection, according to the presence of a subset of HBVpol_455-63_-specific CD8^+^ T cells able to proliferate after Ag encounter. **(B)** Boxplot graph showing the negative correlation between the intensity of HBVpol_455-63_-specific CD8^+^ T-cell proliferation after Ag-specific *in vitro* challenge and the liver stiffness per year of HBV infection. **(C)** Boxplot graph depicting the frequency of HBV DNA testing >2,000 IU/ml during follow-up in relation to the presence or lack of a reactive HBVpol_455-63_-specific CD8^+^ T cells. APRI, AST to platelet ratio index; HBV, hepatitis B virus; pol, polymerase. ^‡^Mann–Whitney U test. ^†^Spearmen correlation test.

According to the HBV transmission risk factor, the point of primoinfection and the length of HBV infection was estimated. In cases without a clear history of HBV exposure antecedent, horizontal transmission during childhood was considered, since our cohort was from Mediterranean origin. In the Mediterranean basin, the main mechanism of transmission is horizontal during early childhood (before the age of 5 years), leading to the development of chronic infection in >50% of cases (12). To calculate the rate of liver fibrosis progression, the ratio between APRI score or LS to the estimated duration of HBV infection was calculated. We observed a significantly lower APRI/duration of HBV infection ratio in those cases with HBVpol_455-63_-specific CD8^+^ T cells with expansion ability (0.004/year) relative to the cohort without proliferation capacity (0.007/year) (**Figure 4A**). Likewise, we detected a lower LS/duration of HBV infection ratio in cases with reactive HBVpol_465-63_-specific CD8^+^ T cells after Ag encounter (0.08 kPa/year) with respect to those cases without expanding cells (0.13 kPa/year) (**Figure 4A**). Furthermore, we observed a significant negative correlation between the intensity of HBVpol_455-63_-specific CD8^+^ T-cell proliferation (% of CD8^+^/pentamer^+^ out of total CD8^+^ cells) and the rate of LS per year of HBV infection (**Figure 4B**). In addition, those cases without a reactive HBVpol_455-63_-specific CD8^+^ T-cell response displayed a higher frequency of HBV DNA tests with a result above 2,000 IU/ml during follow-up (**Figure 4C**).

### 3.4 HBsAg level change during follow-up

**Figure 5:**
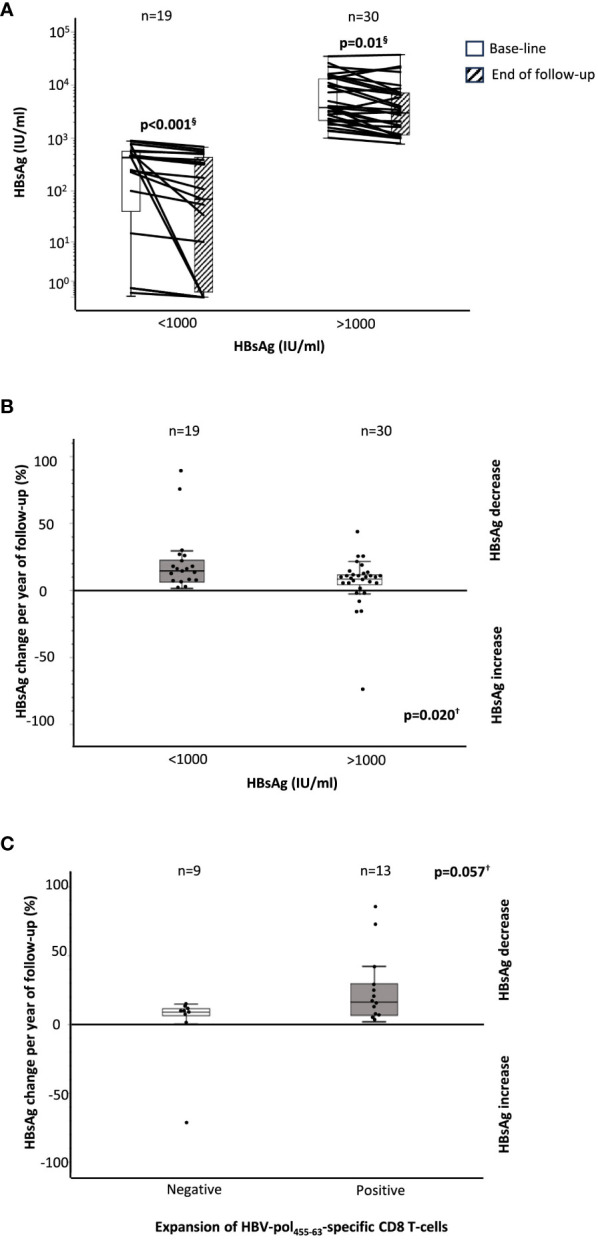
HBsAg decline during follow-up according to baseline HBsAg level and the presence of a functional HBVpol-specific CD8 T-cell response. **(A)** Boxplots showing the variation in HBsAg level between baseline and end of follow-up, according to HBsAg level at recruitment. Boxplots depicting the percentage change in the HBsAg level at the end of follow-up, with respect to the baseline level, **(B)** according to the HBsAg value at recruitment, and **(C)** according to the presence of functional HBVpol-specific CD8^+^ T cells with expansion ability after Ag encounter. HBV, hepatitis B virus; pol, polymerase. ^§^Wilcoxon test. ^†^Mann–Whitney U test.

In 49 cases, we had longitudinal data on HBsAg level at baseline and at the end of follow-up, with a median follow up of 4 years (IQR, 2.6). We observed a significant decrease in HBsAg with respect to the level at recruitment in both HBsAg groups (**Figure 5A**). We calculated the percentage change in HBsAg per year of follow-up, and we observed a significantly greater annual decline in the group with HBsAg level below 1,000 IU/ml with respect to the group with HBsAg higher than 1,000 IU/ml [14.5% (IQR, 18.6) vs. 8.7% (IQR, 7.7)] (**Figure 5B**). Furthermore, in 22 HLA-A2^+^ cases, we observed an almost significantly faster decrease in HBsAg level in those cases with HBVpol_455-63_-specific CD8^+^ T cells with preserved expansion ability with respect to those without reactive cells [16.2% (IQR 29.6) vs. 8.9% (IQR 9.14)] (**Figure 5C**).

## 4 Discussion

In a previous large longitudinal study that followed up a cohort of HBeAg-negative chronic HBV infection for 13 years, 2% of subjects per year of follow-up developed HBeAg(−) chronic hepatitis B. In that study, multivariate analysis showed that HBsAg level >1,000 IU/ml was linked to an increased risk of developing HBeAg(−) chronic hepatitis B in patients previously diagnosed as inactive carriers (5). In our cross-sectional study, we also observed that in subjects defined as HBeAg-negative chronic HBV infection according to EASL guidelines (2), HBsAg level can discriminate different subgroups of inactive carriers with subtle differences in indirect markers of liver inflammation and liver fibrosis. In our work, cases with HBsAg <1,000 IU/ml showed significantly lower transaminase level, APRI score, and liver stiffness and faster HBsAg decline than subjects with HBsAg level >1,000 IU/ml after 5-year median follow-up. In addition, we recorded platelet count as a surrogate of portal pressure and observed a significant lower platelet count in the group with HBsAg >1,000 IU/ml. Similarly, a low platelet count has been described in HBeAg(−) patients with normal transaminases linked to the presence of significant fibrosis (13). We should emphasize that all these parameters assessed were within the normal range in all groups in our cohort, but in any case, these differences could suggest a subtly higher degree of liver inflammation and fibrosis in at least some subjects in the HBsAg >1,000 IU/ml group. We should also note that we reduced the possibility of bias in the estimation of liver fibrosis or inflammation due to potential presence of steatohepatitis along with HBV infection by strict inclusion criteria that excluded cases with high BMI and patients with harmful alcohol intake.

On the other hand, in the highest HBsAg level group, we also observed a higher HBV DNA level at recruitment, which could be related to a worse disease progression. In fact, a recent systematic review described the prevalence of mild inflammatory activity and moderate fibrosis to be approximately 1% among those inactive carriers with HBV DNA levels <2,000 IU/ml but between 7% and 10% among cases with HBV DNA levels between 2,000 and 20,000 IU/ml (14). Moreover, cases with HBV DNA>2,000 IU/ml develop chronic hepatitis in approximately 6% after a median follow-up of 8 years (15). Even in cases with HBV DNA level below 2,000 IU/ml, subjects with HBsAg >1,000 IU/ml are at higher risk of hepatocellular carcinoma and chronic hepatitis than the group with low HBsAg level (5). Therefore, HBeAg-negative chronic HBV infection could be considered as a heterogeneous group that, in a subset of cases, could benefit from treatment because of the potential risk of progression. Actually, the combination of HBsAg level <1,000 IU/ml plus an HBV DNA level <2,000 IU/ml has shown a diagnosis accuracy of HBeAg-negative chronic HBV infection of 94.5%, similar to that of 1-year monthly monitoring (16). Nevertheless, about two-thirds of HBeAg-negative chronic HBV infection have an HBsAg level >1,000 IU/ml (17). In this subgroup, we probably need more tools to detect those patients in risk of progressive liver damage, taking into account that the cutoff value considered as normal transaminases could be inappropriate in some patients (18) and that the presence of significant fibrosis has been reported in up to 20% of HBsAg-positive patients with normal transaminases (19). Other viral markers, such as HB core related Ag (HBcrAg), could play a role to distinguish HBeAg-negative chronic HBV infection cases in risk of progression. HBcrAg optimally discriminates between HBeAg(−) chronic hepatitis B and HBeAg-negative chronic HBV infection (20–22) but also could stratify the risk of fibrosis progression in patients with intermediate viral load (23). Both HBsAg and HBcrAg data suggest that the level of HBV antigenemia might determine the progression of liver damage in HBeAg-negative chronic HBV infection, and this fact could be linked to the effect of antigen load on T-cell function (24, 25).

A functional, multispecific CD8^+^ T-cell response correlates with HBV control during acute hepatitis (26), maintains the inactive carrier state during persistent HBV infection (3), and appears necessary to achieve functional cure after treatment withdrawal of nucleos(t)ide analogues (6, 27). Therefore, the clinical phenotypes described in HBeAg-negative chronic HBV infection in this study could be related to the quality of HBV-specific CD8^+^ T-cell response, which could be modulated according to the antigen level. Actually, in low HBsAg group, we could assume a low level of other HBV antigens, such as env and pol. We observed that the CD8^+^ T-cell proliferative ability after Ag encounter was abolished against HBV-surface Ag and maintained against HBV-core epitope in most of the cases regardless of the HBsAg level. However, CD8^+^ T-cell reactivity against pol was abolished in ≈70% of patients in the high HBsAg level group, whereas it was preserved in most subjects in the low HBsAg level cohort. IFNγ secretion after Ag encounter correlated positively with HBsAg level in HBVcore- and HBV-pol-specific CD8^+^ T cells. TNFα also positively correlated with HBsAg level in HBVcore-specific cells but negatively in HBVpol-specific CD8^+^ T cells. These data suggest that cases with an elevated HBsAg level may have a greater degree of immune activation, visible against HBVcore but impaired against HBVpol, characterized by a weakened proliferation and TNFα secretion tested on T-cell culture supernatant by ELISA. We previously demonstrated that this strategy of cytokine analysis was reliable, since we showed a positive correlation between supernatant cytokine level, tested by ELISA, and cytokine mean fluorescence intensity after intracellular cytokine staining checked by flow cytometry on HBV-specific CD8^+^ T cell after 10-day Ag-specific cultures in HBeAg(−) chronic hepatitis B (6). Nevertheless, since cytokine data were not obtained directly *ex vivo*, these data should be interpreted cautiously because they could also come from re-programmed T cells or from bystander activation.

Overall, we observed a lack of reactive HBVpol-specific CD8^+^ T-cell response in 65% of the cases in the HBsAg>1,000 IU/ml group, which is a frequency similar to that of the reported “gray-zone” cases with significantly histological disease (28). Recently, Schuch et al. demonstrated that in HBeAg(−)-persistent HBV infection with low replicative level, HBVcore- and HBVpol-specific CD8^+^ T cells showed a different level of exhaustion. HBVpol-specific CD8^+^ T cells displayed a more exhausted phenotype and lower functionality than HBVcore-specific CD8^+^ T cells. These authors showed that the impaired proliferative ability of HBVpol-specific CD8^+^ T cells was linked to an imbalanced TCF1/Bcl2 expression (7). In our HBeAg-negative chronic HBV infection cohort, we also observed that CD8^+^ T cells targeting different HBV epitopes exhibited differences in functionality, but only restricted to the group with elevated HBsAg level. One possibility could be that the impaired function of HBVpol-specific CD8^+^ T cells was mainly due to the different amounts of viral antigen. In addition, the length of infection could be involved in the cause of HBV-pol-specific CD8 T-cell exhaustion (29). Another possibility to explain the observed difference could be related with the initial CD8^+^ T-cell priming process (30), which could be impaired for polymerase in a subset of inactive carriers who maintain an elevated HBsAg level. Although HBsAg is not a perfect subrogate of antigenic pressure in HBeAg(−) chronic HBV infection, since HBs gene early integrates in host’s genome (31), we can assume a low quantity of other HBV proteins in low HBsAg patients. In cases with high HBsAg level, this could be either due to the HBs gene integration into host’s genome or owing to the infection of high number of hepatocytes, and in this case, other HBV proteins could also be highly expressed. In those HBeAg(−) chronic HBV infection cases with high HBsAg, the quality HBVpol-specific response could differentiate between those patients with low and high antigenic pressure.

In our cross-sectional study, we observed that cases without HBVpol-specific CD8^+^ T cells might have a subtly faster liver fibrosis progression, assuming that inactive carriers could have a linear fibrosis progression at slow rate as has been described for slow progressors in chronic hepatis C (32). In fact, in a paired liver elastography study carried out in HBeAg-negative chronic HBV infection with HBV DNA ≤ 20,000 IU/ml followed up for 4 years, approximately 15% of patients had either to start treatment or develop fibrosis progression (33). However, the estimated rate of fibrosis progression in our study was very low in all groups and should be confirmed in a longitudinal study. According to our estimation, cases without reactive HBVpol-specific CD8^+^ T cells could have an increase in LS of 1.3 kPa per decade of follow-up, which could mean the development of significant fibrosis after a long-term infection, such as three decades. Moreover, the calculated rate of liver fibrosis progression was inversely related to the intensity of HBVpol-specific CD8^+^ T-cell proliferation after Ag encounter. On the other hand, cases without HBVpol-specific CD8^+^ T-cell reactive response were also more likely to have HBV DNA replication peaks above 2,000 IU/ml during follow-up. This finding could be related with fibrosis progression as what occurs in chronic hepatis B under treatment, in which the presence of persistent low HBV DNA level promotes fibrosis progression despite of therapy (34). In the HBeAg(−) chronic HBV infection, HBsAg level decreases in a biphasic trend, with early continued steady decline and greater drop after 60–70 years of age (35). This decline could be affected by the quality of the adaptive immune response. In fact, we observed an almost significantly slower HBsAg decline in those cases without functional HBVpol-specific CD8^+^ T cells. These tenuous differences between inactive carriers with and without functional HBVpol-specific CD8^+^ T cells might have a minor incidence in the natural history of HBV infection, although it might also help to explain the higher incidence rate of development of chronic hepatitis among inactive carriers with elevated HBsAg level (5).

In summary, we have described different clinical phenotypes in HBeAg(−) chronic HBV infection according to HBsAg level that are related to the functionality of the HBVpol-specific CD8^+^ T-cell response. A subset of inactive carriers with an HBsAg level > 1,000 IU/ml shows indirect data of subtly increased liver inflammation, fibrosis, and portal pressure related to the lack of a functional peripheral HBVpol-specific CD8^+^ T-cell response. Probably, in HBeAg(−) chronic HBV infection with high HBsAg level and absence of HBVpol-specific CD8^+^ T-cell response, further evaluation should be carried out to rule out the need for treatment (15, 28).

## Data availability statement

The raw data supporting the conclusions of this article will be made available by the authors, without undue reservation.

## Ethics statement

The studies involving humans were approved by the Research Ethical Committee of the Guadalajara University Hospital (Spain). The studies were conducted in accordance with the local legislation and institutional requirements. The participants provided their written informed consent to participate in this study.

## Author contributions

JP: Conceptualization, Data curation, Formal Analysis, Investigation, Resources, Writing – original draft, Writing – review & editing. HC: Conceptualization, Data curation, Formal Analysis, Resources, Writing – review & editing. JM: Resources, Writing – review & editing. ES: Resources, Writing – review & editing. AG: Investigation, Writing – review & editing. AD: Data curation, Writing – review & editing. MT: Formal Analysis, Writing – review & editing. JL: Conceptualization, Data curation, Formal Analysis, Funding acquisition, Methodology, Project administration, Resources, Software, Supervision, Writing – original draft, Writing – review & editing.
